# Enhanced Isotopic Ratio Outlier Analysis (IROA) Peak Detection and Identification with Ultra-High Resolution GC-Orbitrap/MS: Potential Application for Investigation of Model Organism Metabolomes

**DOI:** 10.3390/metabo8010009

**Published:** 2018-01-18

**Authors:** Yunping Qiu, Robyn D. Moir, Ian M. Willis, Suresh Seethapathy, Robert C. Biniakewitz, Irwin J. Kurland

**Affiliations:** 1 Stable Isotope and Metabolomics Core Facility, Diabetes Center, Department of Medicine, Albert Einstein College of Medicine, Bronx, NY 10461, USA; Yunping.qiu@einstein.yu.edu; 2 Department of Biochemistry, Albert Einstein College of Medicine, Bronx, NY 10461, USA; robyn.moir@einstein.yu.edu (R.D.M.); ian.willis@einstein.yu.edu (I.M.W.); 3 Thermo Fisher Scientific, Somerset, NJ 08873, USA; suresh.seethapathy@thermofisher.com (S.S.); rbiniakewitz@yahoo.com (R.C.B.)

**Keywords:** isotopic ratio outlier analysis, GC-Orbitrap/MS, positive chemical ionization, *S. cerevisiae*, in silico fragmentation, unknown metabolite identification

## Abstract

Identifying non-annotated peaks may have a significant impact on the understanding of biological systems. In silico methodologies have focused on ESI LC/MS/MS for identifying non-annotated MS peaks. In this study, we employed in silico methodology to develop an Isotopic Ratio Outlier Analysis (IROA) workflow using enhanced mass spectrometric data acquired with the ultra-high resolution GC-Orbitrap/MS to determine the identity of non-annotated metabolites. The higher resolution of the GC-Orbitrap/MS, together with its wide dynamic range, resulted in more IROA peak pairs detected, and increased reliability of chemical formulae generation (CFG). IROA uses two different ^13^C-enriched carbon sources (randomized 95% ^12^C and 95% ^13^C) to produce mirror image isotopologue pairs, whose mass difference reveals the carbon chain length (n), which aids in the identification of endogenous metabolites. Accurate m/z, n, and derivatization information are obtained from our GC/MS workflow for unknown metabolite identification, and aids in silico methodologies for identifying isomeric and non-annotated metabolites. We were able to mine more mass spectral information using the same *Saccharomyces cerevisiae* growth protocol (Qiu et al. Anal. Chem 2016) with the ultra-high resolution GC-Orbitrap/MS, using 10% ammonia in methane as the CI reagent gas. We identified 244 IROA peaks pairs, which significantly increased IROA detection capability compared with our previous report (126 IROA peak pairs using a GC-TOF/MS machine). For 55 selected metabolites identified from matched IROA CI and EI spectra, using the GC-Orbitrap/MS vs. GC-TOF/MS, the average mass deviation for GC-Orbitrap/MS was 1.48 ppm, however, the average mass deviation was 32.2 ppm for the GC-TOF/MS machine. In summary, the higher resolution and wider dynamic range of the GC-Orbitrap/MS enabled more accurate CFG, and the coupling of accurate mass GC/MS IROA methodology with in silico fragmentation has great potential in unknown metabolite identification, with applications for characterizing model organism networks.

## 1. Introduction

Metabolomics has been widely used in disease and model organism research [1,2,3,4]. A major challenge for the field of metabolomics is the identification of unknown or novel metabolites. Mass spectrometry has been significantly increased in terms of sensitivity and resolution over the past decade, however, while in general, tens to hundreds of thousands m/z features (ions) be detected by MS methods, many metabolites are not identified. It is estimated that rarely more than 30% of the compounds are identified in ESI MS/MS untargeted metabolomics profiling [5]. Overall, tandem mass spectral databases are thought to cover less than 1% of the 50 to 90 million compounds catalogued in ChemSpider or PubChem, respectively [5]. Therefore, in general, only a fraction of the data that an untargeted mass spectrometric experiment provides can aid the formation of metabolic networks from which biological conclusions can be made. Challenges for global profiling including the creative use of algorithms for the separation of peaks from noise, optimal data mining paradigms and databases [1].

Recently, Isotopic Ratio Outlier Analysis (IROA) has been developed to enable the characterization of carbon information in a given metabolites or a fragment [6,7,8,9,10]. Unlike other stable isotope labeling methods, rather than utilizing substrates with natural abundance (1.1% of ^13^C isotopomer seen in carbon atoms in nature) and 98–99% enrichment for the control and experimental populations, respectively [11,12,13,14,15], IROA with prototrophic yeast uses randomized 95% ^12^C glucose (5% ^13^C), and 95% randomized ^13^C glucose (5% ^12^C) as carbon sources. This strategy leads to more predictable and diagnostic patterns for the observable isotopic peaks in the mass spectra. The promise of IROA for metabolic phenotyping has been demonstrated in model organism studies. *Saccharomyces cerevisiae*, a prototrophic wild-type strain in the CEN.PK background [16] was grown in minimal yeast nitrogen base (YNB) media, containing either randomized 95% ^12^C, or 95% ^13^C glucose as the single carbon source, in order that the isotopomer pattern of all metabolites would mirror the labeled glucose [10], a protocol which can easily be adapted for microbial species studies. The abundance of the light isotopologues in the 95% ^13^C samples (M_n−1_, M_n−2_, etc., the ^13^C envelope) or the heavy isotopologues in the 95% ^12^C samples (M_0+1_, M_0+2_, etc., the ^12^C envelope), follows the binomial distribution for ^13^C, based on the initial substrate enrichment, in the metabolite products generated. The mass difference between the ^12^C (M_0_) isotopic peak and the ^13^C (M_n_) isotopic peak indicates the number of carbons (*n*) in the metabolite’s carbon backbone. This narrows possibilities for chemical formula generation (CFG), and for normalization between control (^13^C) and treated (^12^C) groups [9,10].

The accurate mass IROA-GC/MS protocol developed, using both chemical ionization (CI) and electron ionization (EI), has been formulated as an algorithm [10], in which the number of carbons, as well as the number of methoximations and silylations are used as search constraints. An accurate mass CI-IROA library with retention times based on the Fiehn protocol has been published [10], where we identified 126 IROA peak pairs, representing 82 annotated and 44 non-annotated metabolites, in prototrophic *S. cerevisiae*.

Another technical improvement for metabolite identification are the in silico fragmentation methodologies [17]. In silico fragmentation software packages developed for prediction of ESI MS/MS spectra include CFM-ID [18], MassFrag [19], MS-Finder [20], and Mass Frontier [21]. Blaženović et al. tested four different in silico fragmentation software packages for the Critical Assessment of Small Molecule Identification (CASMI) data analysis challenge. The results indicate the importance of in silico fragmentation evaluations for metabolite identification. While pure in silico algorithms only identified 17–25% of the compounds correctly, once the chemical database and MS/MS search databases were added, the algorithms were able to correctly identify 87–93% of the compounds in the challenge vs. 60% without in silico evaluation [5]. The development of in silico fragmentation software has mainly focused on LC-MS/MS data, but has recently been applied to high-resolution GC/MS data [22]. CFM-ID [18] can accommodate GC/MS EI data, and can use a HMDB GC/MS database as the GC-EI library, along with the (CI) molecular ion for formulae generation and structural ID.

In this study, we employed the same IROA *S. cerevisiae* protocol, taking advantage of the ultra-high resolution GC-Orbitrap/MS, and we found the number of IROA peak pairs almost doubled when compared to our previous method. We further utilized an in silico fragmentation technique, utilizing CFM-ID to tentatively annotate unknown metabolites, and validated the predictions using procured metabolite standards.

## 2. Results

### 2.1. Choice of Reagent Gas Is Critical for Optimal IROA Pair Detection

In this study, we compared low concentration (5 or 10%) ammonia in methane, pure methane, and pure ammonia as the reagent gas for chemical ionization (CI). The CI reagent gas available for the GC-Orbitrap IROA experiment was 10% ammonia in methane, as opposed to the 5% ammonia in methane we reported as used for GC-TOF/MS IROA determinations [10]. Both concentrations of ammonia, 5% or 10%, in methane as the CI reagent gas generated similar fragmentations for representative metabolites (see Figure S1). However, it has been shown that a higher percentage of ammonia in methane may decrease the ionization efficiency for some functional groups, clearly evident for 20% ammonia in methane [23]. It has been shown that 5% ammonia in methane as reagent gas in positive chemical ionization generates similar mass ions with higher sensitivity as compared with pure ammonia [24], and 10% ammonia in methane as a CI reagent gas is also commonly used [25].

**Figure 1:**
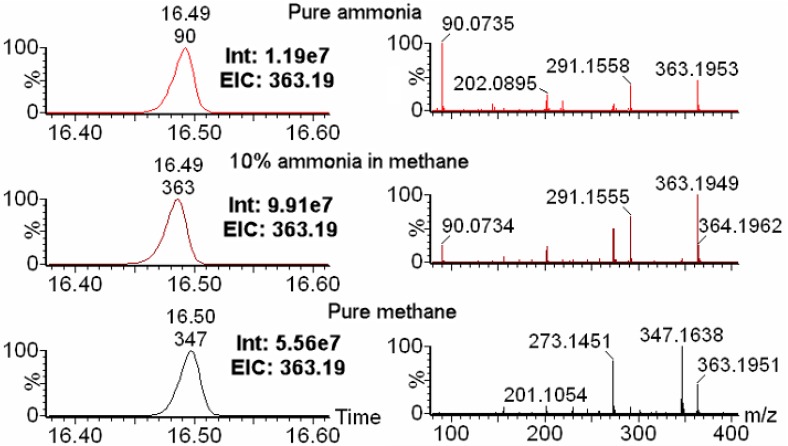
Comparison among three different CI reagent gases. Three reagent gases (pure ammonia (top in red color), 10% ammonia in methane (middle in purple color), and pure methane (bottom in black color)) in positive CI were compared for analysis with unlabeled *S. cerevisiae* extract samples. The left panels are the extracted ion chromatograms (EIC) for the [MH]^+^ ion of triple-silylated glutamine with m/z of 363.195. 10% ammonia in methane showed highest intensity (Int) of the EICs. The right panels are mass spectra of glutamine under three different reagent gases. Pure ammonia showed highest intensity artifact m/z peak of 90.0735, pure methane showed highest intensity of MH-CH_4_ fragment ion (347.1638), while 10% ammonia in methane showed highest intensity of [MH]^+^ ion of triple-silylated glutamine (363.1949).

As shown in the left panels of Figure 1, the extracted ion chromatogram of the [MH]^+^ ion of triple-silylated glutamine (m/z = 363.195) showed highest intensity with 10% ammonia in methane for the same sample. The intensity for 10% ammonia in methane is 9.97 × 10^7^, which is 8.3 times as with pure ammonia as the reagent gas, and 1.8 times as pure methane as the reagent gas. The right panels revealed different mass spectra generated from these three different reagent gases. Pure ammonia generated a high intensity of m/z of 90.0735 (Figure 1), which is a non-biological artifact molecule formed from the silylation group (-SiC_3_H_9_, exact mass = 73.0474) with ammonia (NH_3_, exact mass = 17.0265). This artifact also showed up in the 10% ammonia in methane with much lower intensity compared to the molecular ion of [MH]^+^ for triple-silylated glutamine (MH^+^, m/z = 363.1949). The mass spectra generated by pure ammonia and 10% ammonia in methane are similar. They both showed a higher relative intensity of the [MH]^+^ ion peak than pure methane (m/z = 363.1951), and a dominant fragment of 291.1555, which is due to cleavage one silylation group from the molecular ion (Figure S2). Pure methane as the reagent gas generated a highest intensity of MH-CH_4_ fragment ion (m/z = 347.1638 for triple-silylated glutamine), which is one of the causes for the lower intensity of the m/z of 363.1953 ([MH]^+^) ion when pure methane is used as the chemical reagent gas. In addition to the m/z of 347.1638, pure methane also generated high intensity of the fragment with m/z of 273.1451, which is due to cleavage of an oxygen group and a silylation group (Figure S2). 10% ammonia in methane as the reagent gas also generated a visible, but low intensity, MH-CH_4_ fragment ion, while pure ammonia did not. A much higher intensity of the m/z 273.1451 fragment was seen in the 10% ammonia in methane data compared with that in pure ammonia data.

### 2.2. The Wider Dynamic Range of GC-Orbitrap/MS Allows for Increased Detection of IROA Pairs

There is a noticeable saturation issue for Waters GC-TOF/MS analysis. When the intensity of the signal reaches 1.8 × 10^4^, it saturates under our MS settings used in our previous publication [10]. The dynamic range for Exactive GC-Orbitrap/MS is claimed to be 10 × 10^6^. To obtain more low abundance metabolites, 5 fold more material (1 mL *S. cerevisiae* extract) was used for GC-Orbitrap/MS as compared with the Waters GC-TOF/MS (0.2 mL *S. cerevisiae* extract). The comparison of dynamic range between GC-TOF/MS and GC-Orbitrap is not determined by the number of ions in the starting material. For the GC-Orbitrap dynamic range will be dependent on the automatic gain control and acquisition rate settings of the Orbitrap. Resolution is user settable on the Orbitrap system. In our studies, the resolution was set to 60,000 (at 200 m/z). Based on the resolution setting, the system determines the scan rate. At 60,000 resolution, the scan rate was approximately 7 Hz. The automatic gain control was for 1,000,000 ions with a maximum collection time of 119 ms. The number of ions injected into Orbitrap for analysis is determined by the amount of time (the “injection time”) that the “C” trap has collected ions coming from the quadrapoles. The amount of time of the “C” trap collects ions are, in turn, decided by the intensity of the ions determined two scans prior to this. As a peak starts eluting and reaches maximum, the injection time becomes shorter and shorter. Thus, the system ensures that the total number of ions in the Orbitrap’s C-trap do not exceed significantly above 1,000,000. So, irrespective of concentration, the Orbitrap is always filled with approximately the same amount of ions during each scan, which increases the dynamic range for the Orbitrap in comparison to the GC-TOF/MS.

To demonstrate this saturation issue, 5-oxoproline was used as an example (Figure S3). 5-oxoproline has five carbons in its backbone and two silylation groups attached to it. As we used 95% U^12^C glucose in the IROA experiment, the M_0+1_/M_0_ intensity ratio can be calculated from the probabilities for the abundance of ions in these peaks as predicted by the binomial theorem, 5 × (0.05) × (0.95)^4^/(0.95)^5^ = 0.263. Each silylation site will also carryover 8.54% to the M_0+1_/M_0_ ratio [10], since the natural abundance of the ions in every silylation group (-SiC_3_H_9_) are 0.015% for ^2^H, 4.67% for ^29^Si, and 1.1% for ^13^C.

Therefore, the contribution of the derivatization (M_0+1_ silylation isotopologue) to the carryover of the ^12^C M_0_ metabolite isotopologue peak, for double-silylated 5-oxoproline (M_0+1_, m/z = 275), would be 8.54% × 2 × (intensity of M_0_), for a total M_0+1_/M_0_ of 0.43 (M_0_, m/z = 274). However, in the GC-TOF/MS data (bottom of Figure S3), the intensity of (M_0+1_) is almost equal to that of M_0_. In our previous paper, we solved this problem with a split injection (split ratio of 10) for such peaks. The saturation issue was not observed in GC-Orbitrap/MS data even with a five-fold increase in sample concentration.

The increased dynamic range of the GC-Orbitrap/MS resulted in a significant increase in the number of detected IROA peak pairs with the mixture of ammonia and methane as the reagent gas. In our previous publication, we detected 126 IROA peak pairs (molecular ions) using a Waters GC-TOF/MS, with 82 annotated by Fiehn library, our in-house libraries, and NIST library, using retention time and EI spectrum matches. Here, with GC-Orbitrap/MS, we detected 244 IROA peak pairs (molecular ions) in positive CI mode, and annotated a total of 101 IROA peak pairs (Table 1). While a nearly 100% increase of IROA peak pairs (from 126 to 244) was seen, the annotated peaks only had a 23% increase (from 82 to 101) due to the inclusion of more low intensity metabolites, which tend not to have annotations. Low-intensity EI spectra can be affected by noise and artifacts, decreasing the chance for identification. The low ammonia concentration (10%) in methane reagent gas mixture was best for detecting IROA CI pairs. IROA samples were also run on GC-Orbitrap/MS with pure methane as a reagent gas. Only 116 IROA peaks pairs were detected including 46 annotated ones in the assay using pure methane as the reagent gas (Table 1).

Mass accuracy comparison was performed with 55 shared (identified in both GC-TOF/MS and GC-Orbitrap/MS) annotated metabolites with retention time confirmation by Fiehn library and/or our in-house libraries. The average mass accuracy differences for those 55 metabolites were calculated. The results showed that the average mass differences were 32.2 ppm with a standard derivation of 38.8 ppm for the GC-TOF/MS vs. 1.48 ppm with a standard derivation of 1.25 ppm for the GC-Orbitrap/MS (Table 1).

### 2.3. Enhanced Mass Accuracy Is Associated with Increased Metabolite Identification Capability for the GC-Orbitrap/MS

**Figure 2:**
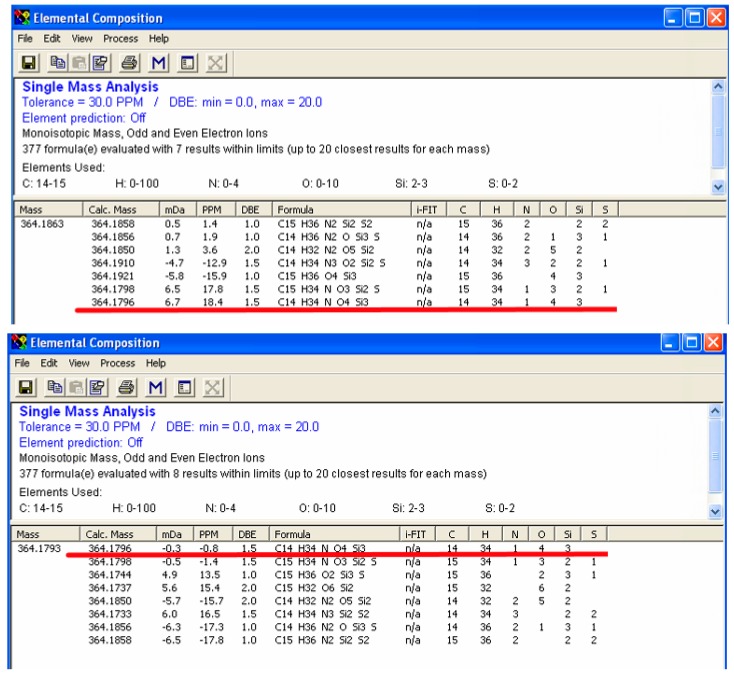
Resolution comparison between GC-Orbitrap/MS and GC-TOF/MS (triple-silylated glutamic acid). Higher resolution helps to reduce possible formulae based on the molecular weight. After considering the number of carbons in the metabolite and silylation groups, the number of carbons was set to 14–15, and the number of silicon atoms was set to 2–3, allowing for possible hydrogen, oxygen, nitrogen and sulfur. The metabolite was identified as triple-silylated glutamic acid with molecular formula as C_14_H_34_NO_4_Si_3_. The accurate mass is 346.1796. The mass derivation for GC-TOF/MS (upper) was 18.4 ppm and ranked as the seventh candidate, while the mass derivation was for GC-Orbitrap/MS (lower) was −0.8 ppm, and ranked as the first of all possible candidates.

Higher resolution is important for unknown metabolite identification, since higher resolution generates a smaller list of CFG possibilities. Here, we used glutamic acid as an example. In the IROA peak pairs (Figure S4), we observed five carbons in its backbone, with m/z of 346 in ^12^C side (M_0_) and 351 in ^13^C side (M_0+n_). The backbone carbon information obtained from GC-PCI-IROA, and the exact mass made it most likely that three silylations present in the metabolite, an estimate supported by the ratio of M_0+n+1_/M_0+n_ [10]_._ The element settings were then selected as follows: carbon, 14–15; hydrogen: 0–100; nitrogen: 0–4; oxygen, 0–10; silylation: 2–3; sulfur: 0–2. Based on our previous experience with GC-TOF/MS [10], the tolerance of mass difference was set to 30 ppm. To show the maximum number of possibility, we used the same mass difference for GC-Orbitrap result. The monoisotopic molecular ion (MH^+^) detected by GC-TOF/MS was 364.1863, while GC-Orbitrap/MS was 364.1793. The exact masses were inputted into elemental composition software (Waters, integrated into MassLynx). The results showed that seven CFGs were suggested in GC-TOF/MS data and the last one is the correct formula for glutamic acid (Figure 2A). It had 18.4 ppm difference between detected monoisotopic molecular ion and the actual one (C_14_H_34_NO_4_Si_3_, Figure 2A). The number of silylation groups was confirmed with BSTFA_d9 derivatization (Figure S4 bottom panel in purple). The elemental composition calculation with GC-Orbitrap/MS data suggested 8 formulae, and the first one is the correct formula for glutamic acid (Figure 2B) with only −0.8 ppm difference between detected monoisotopic molecular ion and the actual one. Based on this result and the results in Table 1, the mass difference for GC-Orbitrap/MS can be restricted to 5 ppm, which significantly lowers the CFGs down to two, with the same order and formulae for the top hits for m/z of 364.1793.

### 2.4. IROA Enhanced Unknown Metabolites Identification with In Silico Fragmentation

**Figure 3:**
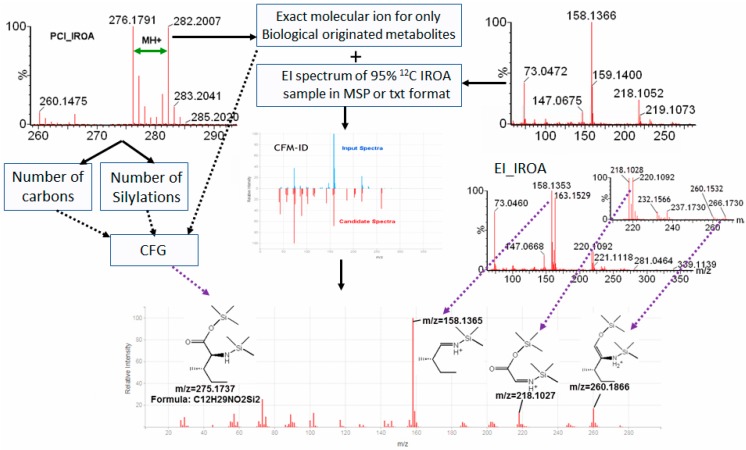
Expanded IROA metabolite identification procedure with in silico fragmentation. Isoleucine is used as an example here. Positive CI-IROA distinguishes unknown metabolites of biological origin from any artificial peaks, and identifies the molecular ion, number of carbons (*n*) in the metabolite from the dalton difference of the M_0_ and M_0+n_ IROA peaks, and number of silylation groups attached to the metabolites. The exact mass of M_0_ molecular ion is combined with the EI mass spectrum found from the 95% ^12^C IROA sample as inputs into CFM-ID to search for potential IDs. The structural identity and its molecular ion can be validated/confirmed from the CI-IROA molecular ion, and the CFM-ID in silico EI fragmentation pattern can be confirmed from the IROA EI pattern.

Using our previous described procedure which combined CI and EI-IROA, and a retention time information match, we could annotate 101 metabolites in 244 IROA peak pairs in this study with the GC-Orbitrap/MS. However, more than hundred non-annotated metabolites of biological origin (metabolites having IROA pairs) are pending for identification. To address this lack, another procedure was developed to include in silico fragmentation into the IROA HR-GC-TOF/MS identification process. Competitive Fragmentation Modeling for Metabolite Identification (CFM-ID, http://cfmid.wishartlab.com/) developed by Wishart’s lab, was employed, as it has the functionality to compare the similarity of EI spectrums with in silico fragmentation [18,22]. We found that IROA aids in the confirmation of CFM-ID predictions. From the CI-IROA patterns, we can select non-annotated metabolites of biological origin for examination by CFM-ID. The exact mass of M_0_ molecular ion of the IROA CI pair is obtained, and combined with EI mass spectrum found from the 95% ^12^C IROA sample as inputs into CFM-ID to search for potential IDs (Figure 3). The deconvoluted EI spectrum in MSP format is inputted into CFM-ID, obtained using AMDIS deconvolution of the EI spectra from the 95% ^12^C sample (see methods). The mass spectrum can be compared with HMDB derivatized database with Jaccard or DotProduct scoring function. The top candidates can be distinguished, and confirmed with the CI and EI-IROA spectra (Figure 3). Firstly, number of carbons and number of silylations from PCI-IROA data can be used to compare with those in the candidates. This, combined with molecular ions, can be used to generate chemical formulae, which can be compared with the chemical formulae of the candidates from CFM-ID. Secondly, the EI IROA spectrum reveals number of carbons for each fragment. In silico fragmentation in CFM-ID can also show the predicted fragments for each mass ion, and the CFM generated from in silico EI fragmentation can be confirmed from the IROA EI patterns. As shown for the example metabolite in Figure 3 (isoleucine), the EI IROA peak pairs of 158–163 (five carbons), 218–220 (two carbons), and 260–266 (six carbons) were able to be elucidated in the in silico fragmentation.

**Figure 4:**
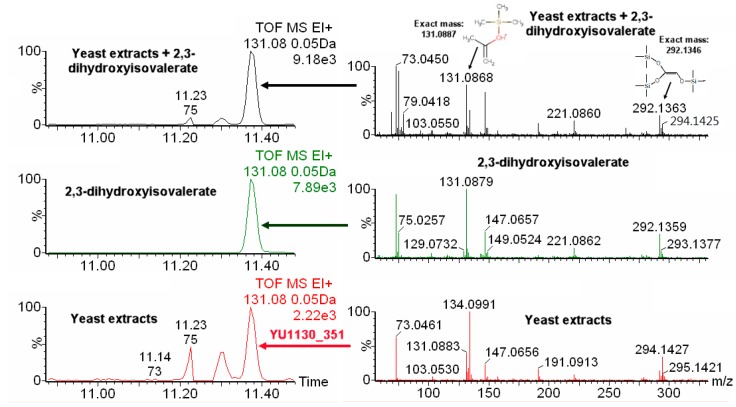
Extracted ion chromatogram (EIC) of m/z 131.08 and mass spectrum of silylated yeast extracts, 2,3-dihydroxyisovalerate, and their combination. Bottom panels (in red color) are the EIC and the mass spectrum from yeast extracts, the middle panels (in green) are the EIC and the mass spectrum from the pure compound (2,3-dihydroxyisovalerate) after derivatization, and top panels (in black) are the EIC and the mass spectrum from yeast extract spiked with pure compound (2,3-dihydroxyisovalerate). The parent molecule has five carbons, and the fragments m/z of 131.0887 and 292.1346 have three carbons and two carbons, respectively, in addition to the silylation group carbons (top right panel). The IROA fragment peak pairs, 131/134 and 292/294, are clearly seen, but their ratio is different between the bottom and top panels due to spiking with the unlabeled 2,3-dihydroxyisovalerate.

The newly-developed procedure was then applied to identify non-annotated metabolites having IROA peak pairs. As an example, we use an unknown IROA peak pair at a retention time of 11.30 that was labeled as YU1130_351 3TMS in our previous publication (see the Supplementary Material section of our previous paper [10]). This compound has five carbons in its backbone. The chemical formula was identified as C_14_H_34_O_4_Si_3_ using our IROA protocol [10]. The EI-IROA spectrum showed two typical IROA peak pairs (131.0883–134.0991, and 292.1345–294.1427, Figure 4, bottom in red color). When the chemical formula was put into NIST MS search, using both the NIST and Fiehn libraries, seven hits were obtained (2-deoxypentofluranose, 3TMS derivative; dimethylolpropanoic acid, 3TMS derivative; D-2Deoxyribose, 3TMS derivative; D-erythro-pentopyrase, 2-deoxy-1,3,4-tri-*O*-(trimethylsilyl)-; deoxyribopyranose, tri(trimethylsilyl) ether (isomer 2); 2-deoxyribose, 3TMS derivative; deoxyribopyranose, tri(trimethylsilyl) ether (isomer 1), Figure S5). However, none of these seven hits has typical mass ions of 131 or 292. Therefore, this metabolite was labeled as an unknown for our in-house library database (see Supplementary Materials section in [10]). The molecular ion (350.1906) and the EI spectrum (see Figure S6, input spectra) from ^12^C IROA sample was inputted into CFM-ID (http://cfmid.wishartlab.com/identify, see Methods). With 50 ppm mass tolerance, 10 candidates were obtained from the HMDB derivatized database, with the metabolite named 2,3-dihydroxyisovalerate (3TMS derivative) on the top (score: 0.0888, Supplement Figure S6). The top five characteristic ions of in silico fragmentation of 2,3-dihydroxyisovalerate (3TMS derivative) with highest intensities are 73, 89, 131, 233, and 335. The ions with 103 and 233 may be the interference from the background (bottom panel, right. Figure 4). We then checked the characteristic ions for in silico fragmentation of five isomeric metabolites (with the same chemical formula of C_14_H_34_O_4_Si_3_ (Figure S7)). CFM-ID generated spectra are most revealing for the EI characteristic ion, which is m/z 131. As shown in Figures S6 and S7, only 2,3-dihydroxyisovalerate and 2,3-dihydroxyvlerate has a 131 m/z EI fragment and an equivalent ranking. Only 2,3-dihydroxyisovalerate was in YMDB database and obtainable for purchase (obtained from Sigma-Aldrich). The spectrum of the authentic standard showed the characteristic ions of m/z 131.0879 and 292.1358 (Figure 4, middle in green color), which have a three-carbon fragment, and a two-carbon fragment, respectively, and EI IROA spectra peak pairs of 131/134 and 292/294. As Figure 4 shows, 2,3-dihydroxyisovalerate had an identical retention time to the unknown. After spiking the standard into the IROA yeast extract, the m/z peaks of the authentic standard exactly overlaps with YU1130_351 (top two panels of Figure 4). The m0 131 and 292 peaks increased in intensity, as did the 221 m/z peak when 2,3-dihydroisovaleric acid was added to the yeast extract (Figure 4, top right panel).

## 3. Discussion

Identifying non-annotated peaks may have a significant impact on the understanding of biological systems. A view has been developing that metabolic networks are more imprecise than precise, that enzymes are not perfectly substrate-specific, and that end products and intermediates of enzyme reactions may undergo spontaneous side reactions [26]. Non-annotated peaks may have compounds generated through metabolite damage and repair [26]. Enzymatic transformations of primary, canonical metabolites, such as by methylation, acetylation, and phosphorylation, can generate active biomolecules that may regulate important cellular and physiological processes [27]. These non-annotated metabolites could provide a regulatory function, as seen for metabolites that are regulators for gene expression through riboswitches in bacteria [28,29]. The Metabolic In silico Network Expansion database (MINE) [30] includes more than a half-million hypothetical compounds, including those derived from potentially reversible reactions, involving single-reaction modifications, such as simple methylations, acetylations, and hydroxylations, which would enable the formation of an epimetabolite. Prior work that developed a systematic workflow for annotating unknown epimetabolites using MINE, in silico fragmentation, and high-resolution GC-QTOF/MS, determined the molecular mass by aligning molecular adduct ions in EI and CI spectra and the number of TMS groups for the exact formulae based on the mass unit shift between compounds derivatized with TMS and TMS-d9 [22]. Our protocol, based on IROA, has the advantages of determining both the number of carbons in backbone of the derivatized metabolite, and the number of TMS groups from CI alone without the need for stable isotope derivatization reagents [10], which have a retention time shift relative to non-deuterated derivatization reagents.

One of the major advantages for IROA technique is to identify metabolites/peaks of biological origin from artificial peaks or backgrounds. As a result, metabolites of biological origin with IROA peak pairs in low concentration could be readily detectable from noise, and distinguished from artifacts, which will not show IROA pairs. As shown in Figure 4, IROA peak pairs 131–134, and 292–294 clearly showed up among other unpaired ions, such as 73, 147, 103, and 221. Using CFM-ID and HMDB we successfully identified this unknown metabolite as 2,3-dihydroxyisoverate, which is not in the commercial GC-MS databases (such as NIST, and Fiehnlib).

In general, for the GC-Orbitrap runs, the C trap filled quickly, so there were low abundance peaks that were detectable, but these peaks were difficult to perform spectral matching and annotation, and we found that extremely low intensity fragmentations may lose their IROA pairs. As in our result, the fragments 335 and 277 did not show up in the IROA samples (which are in the spectrum of authentic 2,3-dihydroxyisovalerate standard, not shown here). In this case, EI IROA pairs of characteristic ions are crucial for metabolite identification and, in the future, an EI IROA library may have use in unknown metabolite identifications.

Here, once the exact mass of the M_0_ molecular ion of the IROA CI pair is obtained, and combined with EI mass spectrum found from the 95% ^12^C IROA sample as inputs into CFM-ID, it is possible to search for potential IDs. The method is designed to determine the correct choice among isomeric metabolites, and is dependent on the unknown structure being in HMDB. If the non-annotated (epi)metabolite is not in any database, the method of Fiehn and co-workers can be used [22] but, again, the workflow can be simplified if IROA can be used for exact formula determination using CI, which is quite possible for some model organism studies.

## 4. Materials and Methods

Yeast culture and extraction are the same as our previous publication [10]. Briefly, a prototrophic wild-type haploid *S. cerevisiae* strain, DBY10085 (Mata; URA; LEU; HIS; TRP; MAL2-8^C^; SUC2 [16]), in the CEN.PK background was grown in 95% ^12^C glucose or 95% ^13^C glucose (IROAtech Inc.) in IROA YNB medium with a final glucose concentration of 2%. Cells were grown in batch culture at 30 °C in a shaking water bath and harvested at early log phase (OD600 of 2.5) with rapid filtration extraction into 1 mL of chilled (−20 °C) methanol/acetonitrile/water (4:4:2 (*v*:*v*:*v*)) [13,31]. The cell extract was transferred to a 2 mL polypropylene tube containing 500 μL of glass beads and physically lysed at 4 °C in a mini beads beater using four cycles of 30 s with a 1 min rest on ice between each cycle. The extract was clarified by centrifugation at 14,000 rpm for 5 min, the supernatant collected, and cell debris back-extracted twice with 500 μL of extract solution. The supernatants were pooled, centrifuged at 14,000 rpm for 5 min and the final supernatant stored at −80 °C.

A volume of 1 mL (for GC-Orbitrap-MS) or 0.2 mL (for Waters GC-TOF/MS) extracted solution was pipetted to a GC vial and dried under speedvac. A two-step derivatization was performed. First, methoxymation was performed by adding 50 µL of methoxyamine hydrochloride (MOX, 15 mg/mL in pyridine) to the dried samples, and kept at 30 °C for 90 min. Then, a silylation reaction was performed by adding 50 µL of *N*,*O*-bis(trimethylsilyl)trifluoroacetamide (BSTFA, containing 1% TMCS), and kept at 70 °C for 60 min.

Metabolites separation was performed with a TR-5MS GC columns (30 m × 0.25 mm × 0.25 µm, Thermo Scientific). One microliter of derivatized samples were injected with splitless mode. Helium was used as carrier gas at a consistent flow of 1 mL/min. Pure methane or 10% ammonia in methane was used as reagent gas in the positive chemical ionization mode at a flow rate of 1.5 mL/min. Orbitrap was run in a full scan mode with the resolution of 60,000 relative to 200 Da (at this resolution setting, the maximum rate is 7 Hz). The AGC gain was set to 10 × 10^6^, and maximum injection time of 119 ms. The mass range was set to 80–1000. The injection temperature was set to 280 °C. The oven program was initiated at 60 °C, kept for 1 min, rise to 320 °C at a rate of 10 °C/min, and kept at 320 °C for 5 min.

The data were processed with Mass Frontier software (Thermo Scientific, Waltham, MA, USA), and MassLynx software (Waters Co., Milford, MA, USA). MSP files were created with AMDIS and NIST MS search software. An EI data file was first inputted into AMDIS software. The corresponding peak was then imported to the NIST MS search and then exported as an MSP file. The MSP file can then be inputted into CFM-ID to search for candidates (http://cfmid.wishartlab.com/identify). SMILE strings for original metabolites (underivatized form) for the candidates can be obtained through HMDB or the PubChem website (https://www.pubchem.ncbi.nlm.nih.gov). The SMILE strings for the original metabolites can be inputted into MetaboloDerivatizer (http://prime.psc.riken.jp/Metabolomics_Software/MetaboloDerivatizer/index.html) to obtain derivatized SMILE strings. Derivatized SMILE strings can then be inputted into CFM-ID to acquire in silico fragmentation (http://cfmid.wishartlab.com/predict), which can be then compared with EI-IROA spectra.

## 5. Conclusions

Using the ultra-high resolution GC-Orbitrap/MS, we re-analyzed *S. cerevisiae* IROA samples generated in our previous study [10]. The GC-Orbitrap/MS had wider dynamic range compared with the Waters Premier GC-TOF/MS, and results in more IROA peak pairs detected. The GC-Orbitrap/MS also provides higher resolution, which increases the reliability of CFG. We also tested three different reagent gases for positive CI. The mixture of a low concentration of ammonia (either 5% or 10%) in methane was proved to be optimal for CI reagent gas for IROA samples, and detected more IROA peak pairs than either pure methane or pure ammonia. The higher resolution and wider dynamic range of the GC-Orbitrap/MS enabled more accurate CFG. We further developed a procedure that coupled this IROA technique with in silico fragmentation and demonstrated, using this complementary combined IROA/in silico fragmentation method, the successful identification of an unknown metabolite, 2,3-dihydroxyisoverate, which is not in the commercial GC-MS databases (such as NIST, and Fiehnlib). The coupling of accurate mass GC/MS IROA methodology with in silico fragmentation has great potential in unknown metabolite identification, with applications for model organisms.

## Figures and Tables

**Table 1:** Overall comparison between IROA results obtained from GC-Orbitrap and GC-TOF/MS, with different CI reagent gases. The GC-Orbitrap allows more material to be injected due to wider dynamic range, which results in the detection of more IROA pairs. Pure methane as reagent gas produces more fragments than using an ammonia/methane mixture, and pure CI methane will lower the intensity of the molecular ion, resulting in less IROA metabolites detected. GC-TOF/MS used 5% ammonia in methane as the reagent gas, and GC-Orbitrap/MS used 10% ammonia in methane as the reagent gas.

	GC-TOF/MS 5% Ammonia in Methane	GC-Orbitrap/MS Methane	GC-Orbitrap/MS 10% Ammonia in Methane
Extracted material used	200 μL	1 mL	1 mL
Saturation in splitless injection	Yes	No	No
IROA peak pairs	126	116	244
Annotated	82	46	101
Average mass difference for 55 metabolites (ppm)	32.2 ± 38.8	-	1.48 ± 1.25

## Supplementary Materials

The following are available online at http://www.mdpi.com/2218-1989/8/1/9/s1, Figure S1: Mass spectra of 10 typical CI-IROA peak pairs with fragments obtained from GC-Orbitrap/MS and GC-TOF/MS. Figure S2: Elucidation of the fragmentations for tri-silylated glutamine for positive chemical ionization with methane, ammonia or the mixture as the reagent gas. Figure S3: Demonstration of the lack of saturation for GC-Orbitrap/MS vs. GC-TOF/MS when metabolites at high concentration (5-oxoproline) are present in the sample. Figure S4: IROA peak pairs for tri-silylated glutamic acid detected in GC-TOF/MS, and in GC-Orbitrap/MS. Figure S5: Demonstration of the representations of isomeric metabolites (seven found) with chemical formula of C14H34O4Si3 in the NIST library. Figure S6: Top 10 hits in CFM-ID with EI spectrum from ^12^C IROA sample, with the peak of YU1130_351. Figure S7: EI in silico fragmentation spectra of the metabolites with chemical formula of C_14_H_34_O_4_Si_3_ in the NIST library.
