# Supplementary material for: Enhanced Isotopic Ratio Outlier Analysis (IROA) Peak Detection and Identification with Ultra-High Resolution GC-Orbitrap/MS: Potential Application for Investigation of Model Organism Metabolomes
Source: Metabolites. 2018 Jan 18;8(1):9. doi: 10.3390/metabo8010009 (PMC5875999; doi:10.3390/metabo8010009)
Supplement: Supplementary file 1 [file metabolites-08-00009-s001.pdf]

Supplementary Materials: Enhanced Isotopic Ratio Outlier Analysis (IROA) peak detection and identification with ultrahigh resolution GC-Orbitrap/MS: Potential Application for Investigation of Model Organism Metabolomes

Yunping Qiu, Robyn D. Moir, Ian M. Willis, Suresh Seethapathy, Robert C. Biniakewitz and Irwin J. Kurland

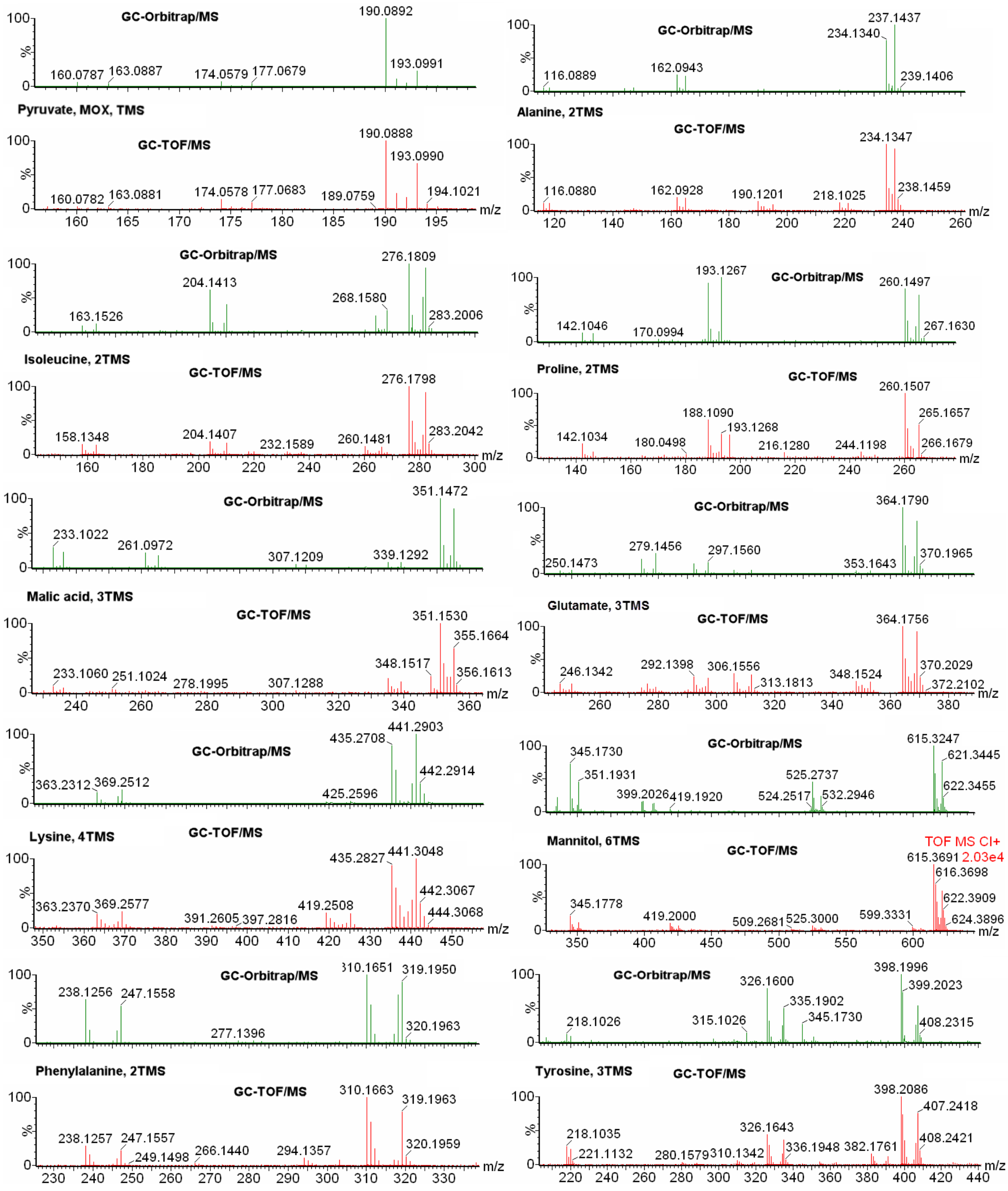

Figure S1. Mass spectra of 10 typical CI-IROA peak pairs with fragments obtained from GC-Orbitrap/MS (on the top of each panel) and GC-TOF/MS (at the bottom of each panel). The reagent gas for both is a low concentration of ammonia in methane, which for the GC-TOF/MS was 5% ammonia in methane, and 10% ammonia in methane for GC-Orbitrap/MS.

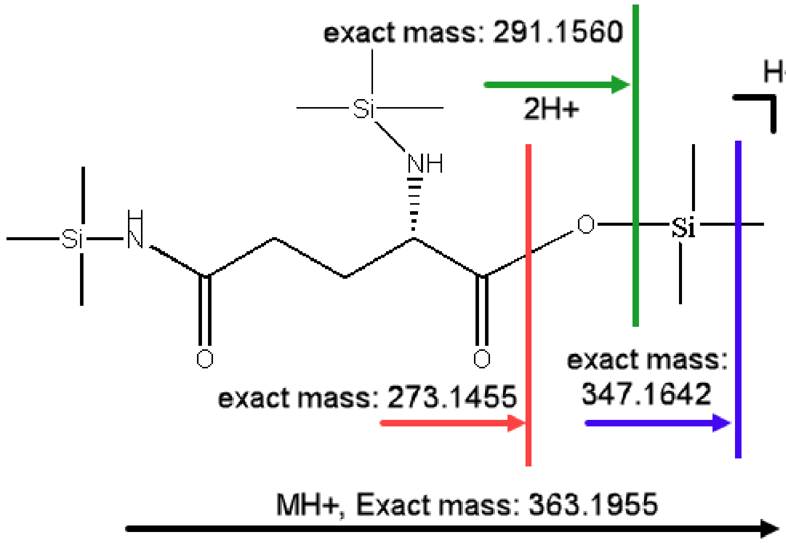

Figure S2. Elucidation of the fragmentations for tri-silylated glutamine for positive chemical ionization with methane, ammonia or the ammonia/methane mixture as the reagent gas. Pure methane as the reagent gas generated a larger intensity MH-CH4 fragment ion (m/z = 347.1638 for tri-silylated glutamine), as well as a high intensity of the fragment with m/z of 273.1451, due to cleavage of an oxygen group with the silylation group attached to it. The mass spectra generated by pure ammonia and 10% ammonia in methane showed a higher relative intensity molecular ion peak (m/z 363.195), and a dominant fragment of m/z 291.155 (due to cleavage one silylation group from the molecular ion).

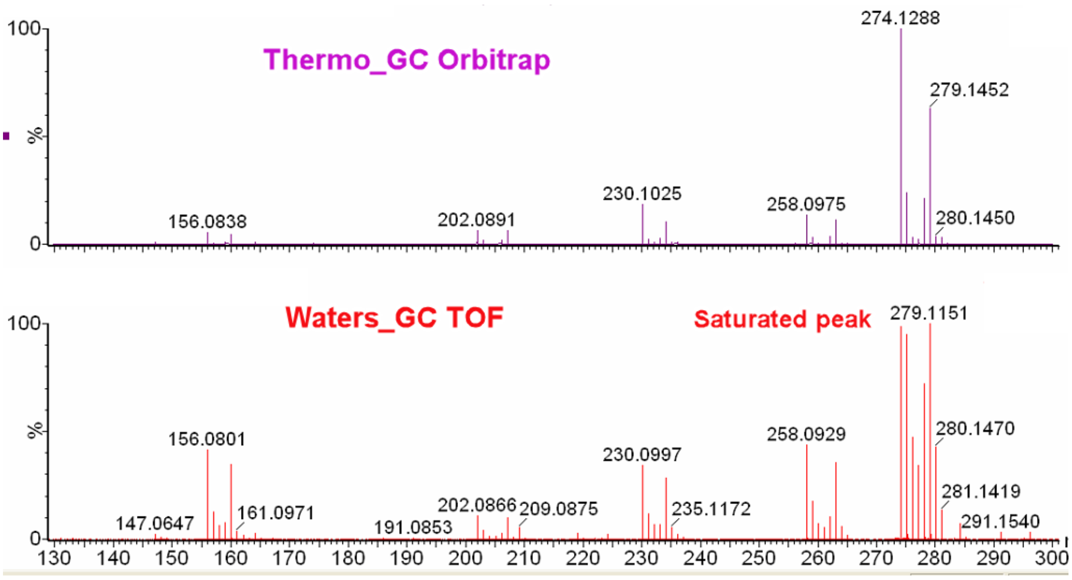

Figure S3. Demonstration of the lack of saturation for GC-Orbitrap/MS *vs.* Waters GC-TOF/MS when metabolites at high concentration (5-oxoproline) are present in the sample. To detect a greater number of lower abundance metabolites, 5 fold more extracted yeast material was used for the GC-Orbitrap/MS as compared with GC-TOF/MS. Even with a lower amount of starting material, saturation was observed in the molecular ion (m/z 274)

in GC-TOF/MS (lower) spectrum. The same IROA PCI fragments (5 carbon m/z 258/263, and m/z 202/207, and 4 carbon m/z 156/160, and m/z 230/234) were observed in both GC-TOF/MS and GC-Orbitrap/MS spectrum.

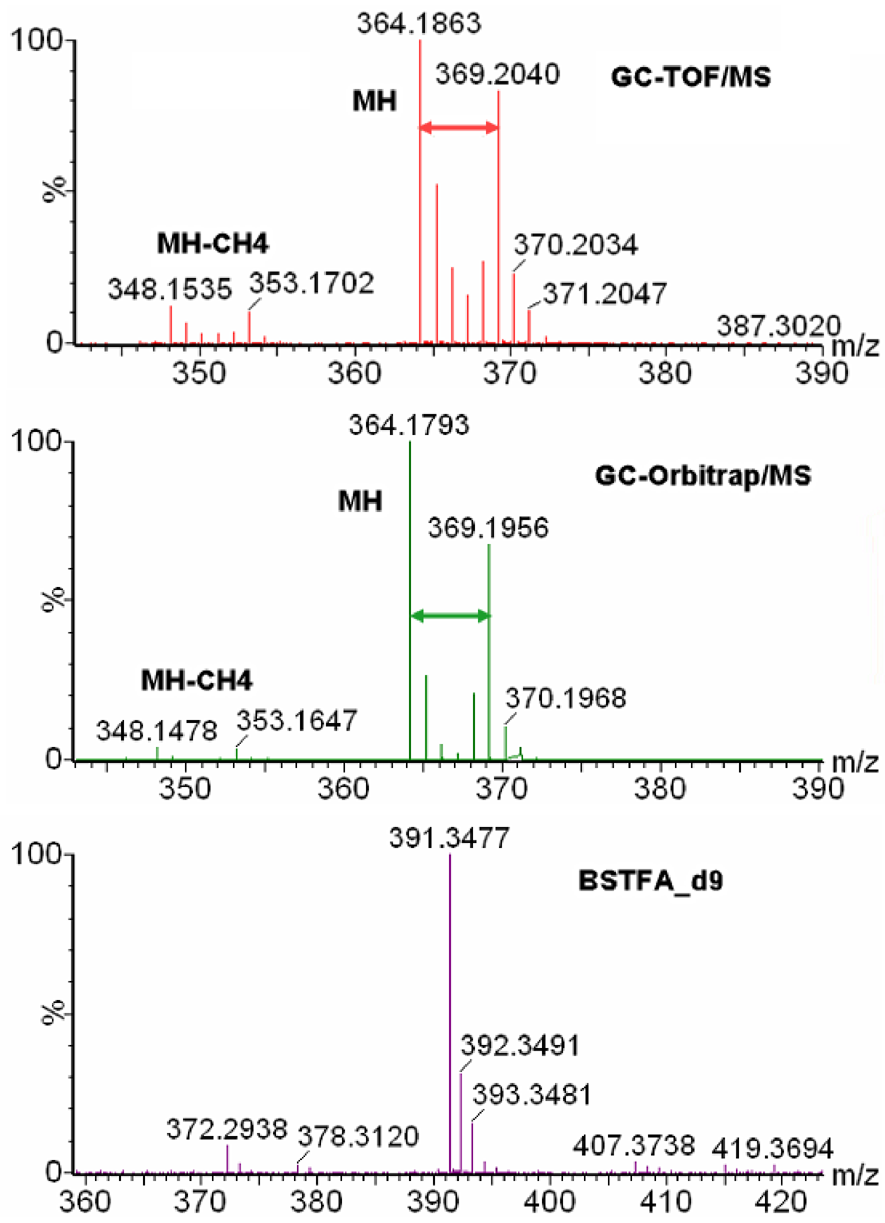

Figure S4. IROA peak pairs for tri-silylated glutamic acid detected in GC-TOF/MS (top in red color), and in GC-Orbitrap/MS (middle in green color). The $[MH]^+$ peak pairs are 364 to 369 m/z (5 carbons). BSTFA_d9 derivatization revealed 3 silylations in glutamic acid with a 27 Da difference between BSTFA_d9 and unlabeled BSTFA derivatization (m/z 364–> m/z 391, bottom panel in purple), validating CFG predictions in Figure 2.

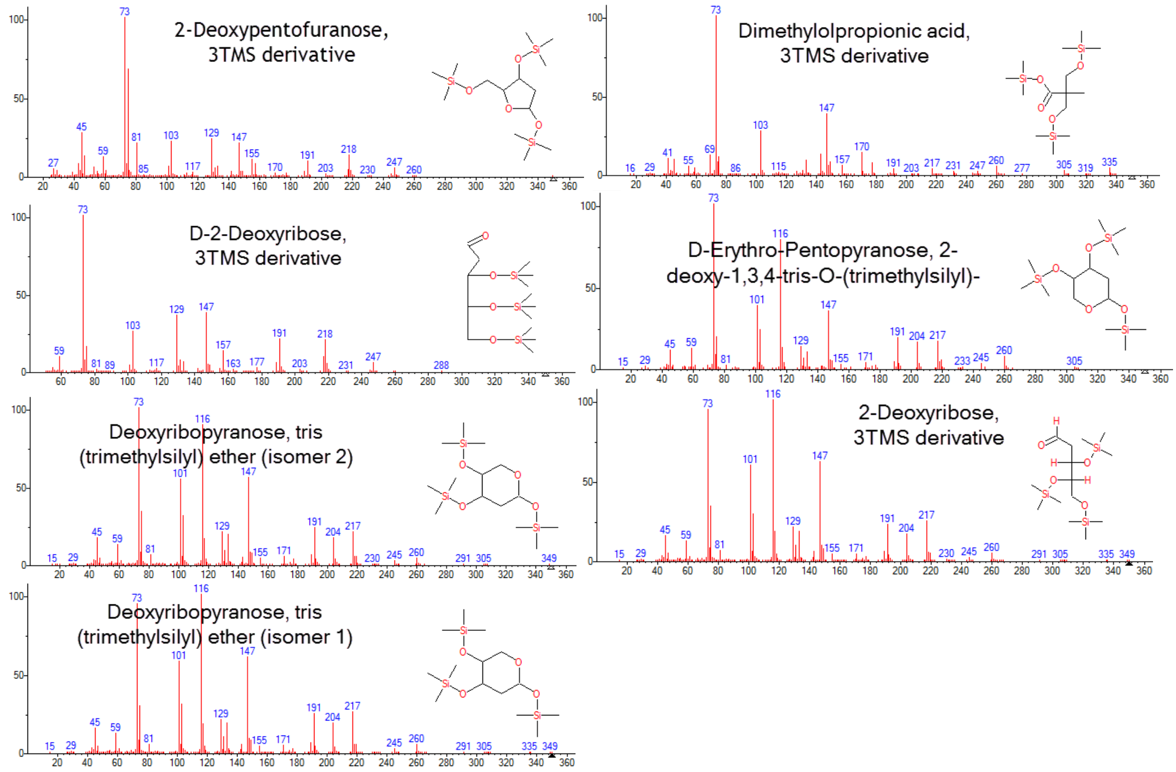

Figure S5. Representations of isomeric metabolites (seven found) with chemical formula of $C_{14}H_{34}O_4Si_3$ in the NIST library.

Predicted MsMs Spectrum (70eV), M+

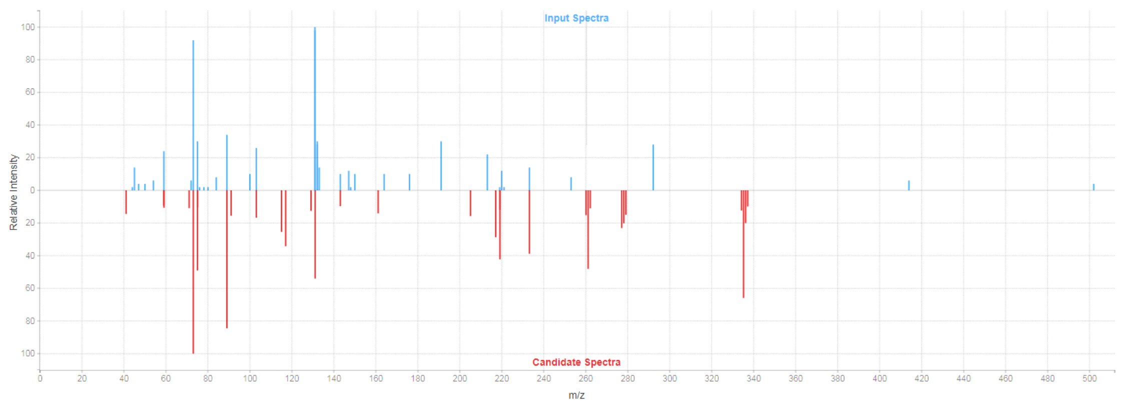

Candidate Rankings

Rank	Score	Structure	ID	Name	Chemical Formula	Mass	Compare
1	0.088767766	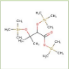	HMDB_DERIV_70144 Original: HMDB12141	(3 TMS) derivative of (R)-2,3-Dihydroxy-isovalerate	C14H34O4Si3	350.17648	Current
2	0.080804117	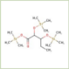	HMDB_DERIV_816 Original: HMDB00421	(3 TMS) derivative of 2,3-Dihydroxyvaleric acid	C14H34O4Si3	350.17648	Compare
3	0.063123681	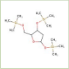	HMDB_DERIV_39507 Original: HMDB03224	(3 TMS) derivative of Deoxyribose	C14H34O4Si3	350.17648	Compare
4	0.043538546	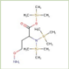	HMDB_DERIV_70745 Original: HMDB12251	(3 TMS) derivative of L-Canaline	C13H34N2O3Si3	350.18771	Compare
5	0.043100425	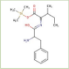	HMDB_DERIV_16301 Original: HMDB28998	(1 TMS) derivative of Phenylalanyl-Isoleucine	C18H30N2O3Si	350.20255	Compare
6	0.036206002	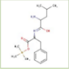	HMDB_DERIV_73873 Original: HMDB13243	(1 TMS) derivative of Leucyl-phenylalanine	C18H30N2O3Si	350.20255	Compare
7	0.035176528	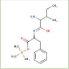	HMDB_DERIV_14831 Original: HMDB28914	(1 TMS) derivative of Isoleucyl-Phenylalanine	C18H30N2O3Si	350.20255	Compare
8	0.031104191	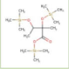	HMDB_DERIV_19707 Original: HMDB29576	(3 TMS) derivative of 2,3-Dihydroxy-2-methylbutanoic acid	C14H34O4Si3	350.17648	Compare
9	0.011789841	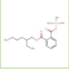	HMDB_DERIV_73887 Original: HMDB13248	(1 TMS) derivative of Monoethylhexyl phthalic acid	C19H30O4Si	350.19132	Compare
10	0.0068566833	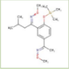	HMDB_DERIV_26452 Original: HMDB32589	(2 MeOx) (1 TMS) derivative of 1-(5-Acetyl-2-hydroxyphenyl)-3-methyl-1-butanone	C18H30N2O3Si	350.20255	Compare

Figure S6. Top 10 hits using CFM-ID with the EI spectrum from the 12C IROA example (YU1130_351, see CI-IROA library in Supplemental section of reference [10]). Settings in CFM-ID: Spectra type: EI; choose a database: HMDB derivative; Parent ion mass: 350.1906; scoring function: DotProduct; with 50 ppm mass tolerance.

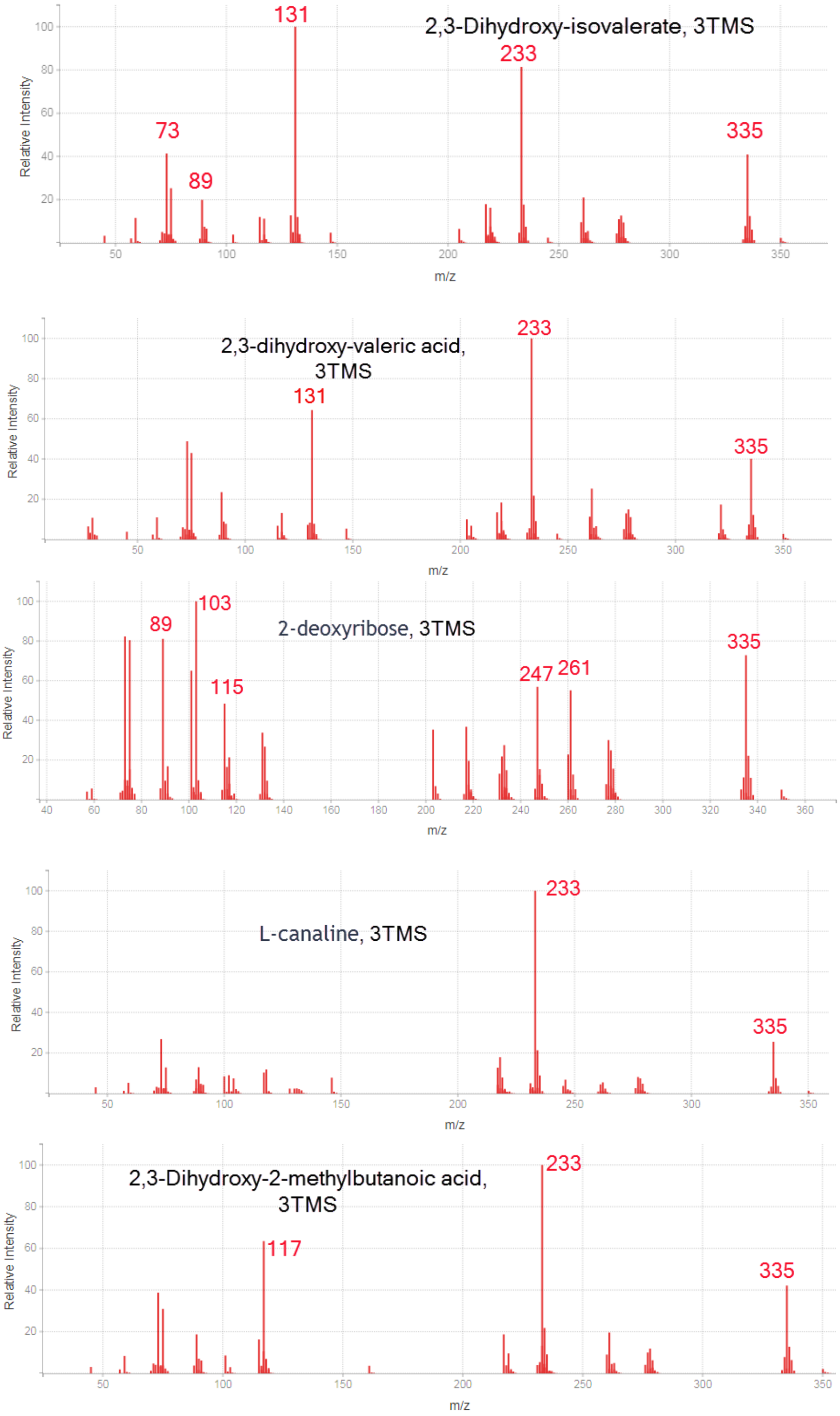

Figure S7. EI in silico fragmentation spectra of the metabolites with chemical formula of $C_{14}H_{34}O_4Si_3$ in the CFM-ID. Note that the dominant theoretical EI peak of m/z 131 is evident is the 2,3-dihydroxyisovaleric acid seen in Figure 4.
